# Structural Insight into the Interactions between Structurally Similar Inhibitors and SIRT6

**DOI:** 10.3390/ijms21072601

**Published:** 2020-04-09

**Authors:** Shuang Zhao, Yan-Yan Zhu, Xiao-Yu Wang, Yong-Sheng Liu, Yun-Xiang Sun, Qing-Jie Zhao, Hui-Yu Li

**Affiliations:** 1 Department of Mathematics and Physics, Shanghai University of Electric Power, Shanghai 20090, Chinayyzhu@shiep.edu.cn (Y.-Y.Z.); wxy94929@163.com (X.-Y.W.); ysliu@shiep.edu.cn (Y.-S.L.); 2 Department of Physics, Ningbo University, Ningbo 315211, China; wlxsunyunxiang@gmail.com; 3 Shanghai Institute of Material Medical, Chinese Academy of Sciences, Shanghai 201203, China

**Keywords:** SIRT6, inhibitor, nature molecule, molecular dynamics simulation, scutellarin, hydrophobic interactions, π–stacking interactions, binding sites

## Abstract

Sirtuin 6 (SIRT6) is an NAD+-dependent deacetylase with a significant role in 20% of all cancers, such as colon cancers and rectal adenocarcinoma. However, there is currently no effective drug for cancers related to SIRT6. To explore potential inhibitors of SIRT6, it is essential to reveal details of the interaction mechanisms between inhibitors and SIRT6 at the atomic level. The nature of small molecules from herbs have many advantages as inhibitors. Based on the conformational characteristics of the inhibitor Compound 9 (Asinex ID: BAS13555470), we explored the natural molecule Scutellarin, one compound of Huang Qin, which is an effective herb for curing cancer that has been described in the Traditional Chinese Medicine (TCMS) library. We investigated the interactions between SIRT6 and the inhibitors using molecular dynamics (MD) simulations. We illustrated that the structurally similar inhibitors have a similar binding mode to SIRT6 with residues—Leu9, Phe64, Val115, His133 and Trp188. Hydrophobic and π-stacking interactions play important roles in the interactions between SIRT6 and inhibitors. In summary, our results reveal the interactive mechanism of SIRT6 and the inhibitors and we also provide Scutellarin as a new potential inhibitor of SIRT6. Our study provides a new potential way to explore potential inhibitors from TCMS.

## 1. Introduction

Sirtuins are a family of NAD^+^-dependent enzymes that have different key roles, such as in aging, metabolism, nutritional behaviour, cancer and inflammation [1,2,3,4]. This family includes seven sirtuins (SIRT1–7) with different enzymatic activities, substrate proteins and biological functions [5]. To date, growing evidence has demonstrated that sirtuins have become increasingly apparent in the pathophysiology of diseases, which brings the possibility of using sirtuin inhibitors at the initial phase of tumorigenesis to fighting cancer [6,7,8]. Owing to the multiple regulatory roles of sirtuins, there are many reports on SIRT1 [9], SIRT2 [10] and SIRT5 [11] inhibitors; however, other sirtuins, such as SIRT6, remain without drugs [12].

SIRT6 is a sirtuin family member that regulates gene expression and plays a role in inflammation, glucose metabolism and genomic stability [13,14,15,16,17,18,19]. All of the roles mentioned above are associated with tumour-initiating, for example, in many cancers, such as oesophageal cancer [20], breast cancer [21] and skin cancer [22]. SIRT6 functions as a tumour promoter. It has also been reported that SIRT6 cooperated with special small molecules is deleted or reduced in 20% of all cancers, such as colon cancers and rectal adenocarcinoma, according to the Cancer Genome Atlas database [17]. Crystal structure of SIRT6 (PDB code 3K35) [23,24] includes the hydrophobic channel, the Rossman fold and the small domain shown in Figure S1A [25]. The Rossman fold is one of the most common and widely distributed super-secondary structures. The dinucleotide such as FAD, NAD and NADP can bind in the domain [26]. There is a long hydrophobic-channel pocket that binds the small molecules [27].

Probing potential inhibitors has become essential for the development of cancer drug design [4,28,29]. Several potential inhibitors of SIRT6 have been reported, such as Compound 9 [3,30], trichostatin A [23] and inhibitors with a salicylate-linked structure [31]. Parenti et al. synthesized three (SYN17739303, BAS13555470 or Compound 9 and BAS00417531) potential inhibitors for SIRT6 [30]. Interestingly we found that the three inhibitors have similar chemical structure. The structures of these inhibitors can give insight into the direction of the design of improved inhibitors. However, the mechanism of interactions between the inhibitors and SIRT6 is still elusive. The relationship between the interactions and the bioactivity of SIRT6 is also not clear.

Small molecules from herbs have many advantages as inhibitors. For example, herbal medicine has low toxicity and can be easily absorbed and easily expelled from the body. In this investigation, we therefore sought to probe structure-based inhibitors similar to Compound 9 [30] from Traditional Chinese Medicines (TCMS). Scutellarin is one compound of *Scutellaria baicalensis* Georgi, named Huang Qin in TCMS. It has been reported that Huang Qing can cure cancer in the Chinese Pharmacopoeia [32]. And recent experimental studies also expressed that a chemically standardized extract from Huang Qing is effective in human colon cancer cell lines [33]. Several clinical and basic studies have shown that flavonoids, including Scutellarin, have significant effects on diabetes [34], obesity [35], cardiovascular diseases [36], cancer [37] and other diseases. However, the effect of Scutellarin on SIRT6 and the underlying mechanism are largely unknown.

In this study, the mechanism of action of small molecules and proteins was explored. We carried out extensive atomistic MD simulations in explicit water of 400 ns in length on the SIRT6 system and 400 ns in total for the SIRT6+Compound 9 and SIRT6+Scutellarin systems. Here, we report the molecular mechanism between SIRT6 and the inhibitors, provide the binding model of the complex systems and compare the two complex systems, thus paving the way towards further development of new SIRT6-targeted inhibitors. Our studies provide a new potential way to explore potential inhibitors from TCMS.

## 2. Results and Discussion

The choice of the natural Chinese herbal molecule Scutellarin. There are many Chinese herbs that have therapeutic effects on the cancer, for example, Huang Qin [33], Ge Gen [38], Bai Shu [39], Ban Zhi Lian [40] and Gan Cao [41]. Therefore, based on the structural characteristics of Compound 9, we tried to explore potential inhibitors that have a similar conformation to compound 9 from TCMS, which has three carbon rings in a line in terms of a 2D chemical structure as shown in Table 1. We chose small molecules with a long alkyl chain or aromatic ring structure from TCMS using AutoDock methods [42] in Table 1. Based on our analysis, we found that Scutellarin has the lowest binding energy with SIRT6, as shown in Table 1. Scutellarin is one component of *Scutellaria baicalensis* Georgi, named Huang Qin in TCMS, which has been described as curing cancers in the Chinese Pharmacopoeia [32].

**Figure 1:**
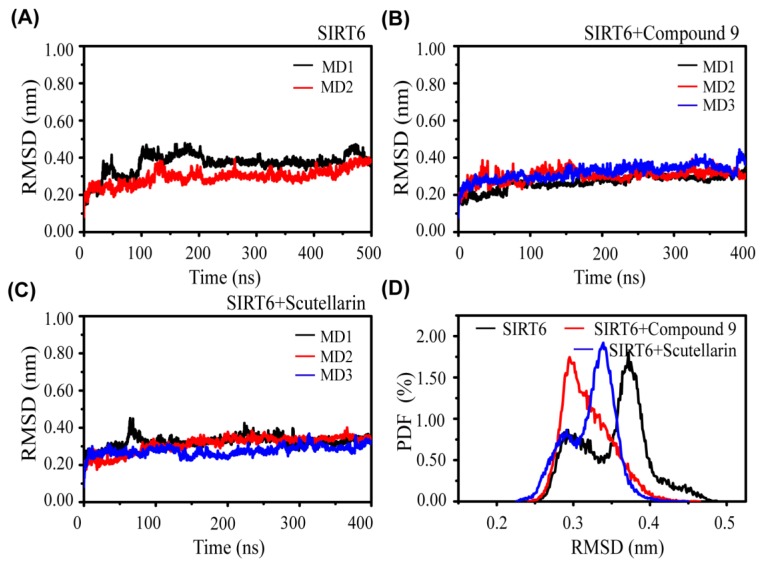
The overall root mean square deviation (RMSD) of SIRT6 with and without the inhibitors. (**A**) SIRT6 system, (**B**) SIRT6+Compound 9 system and (**C**) SIRT6+Scutellarin system. (**D**) The probability distribution function of the RMSD of SIRT6 in the SIRT6 system (black), SIRT6+Compound 9 system (red) and SIRT6+Scutellarin system (blue).

The Convergence and Conformational characteristics of SIRT6 in the three systems. To check the convergence and stability of the simulation systems, we first calculated the Cα–RMSD of SIRT6 in the three systems: SIRT6, SIRT6+Compound 9 and SIRT6+Scutellarin (Figure 1). The RMSD values of SIRT6 in the three systems increased rapidly from the initial value (0 nm) to a value of ~0.3 nm during the first 100 ns of simulation and then reached a plateau during the last 300 ns. This indicates that the simulations reach equilibrium after t = 100 ns. We can see that the RMSD values in the two MD runs of the SIRT6+Compound 9 system and SIRT6+Scutellarin system are approximately 0.35 nm, which is slightly lower than those of the SIRT6 system (0.4 nm). In addition, the two complex systems converge at approximately 100 ns shown in Figure 1B and the SIRT6 system also converges at 200 ns shown in Figure 1A, indicating that the inhibitors stabilize the conformation of SIRT6. 

We further examined the probability of the RMSD from three systems, as shown in Figure 1D. We observed that the peak probability of RMSD was decreased by 0.17 nm and 0.04 nm upon compound binding in the SIRT6+Compound 9 and SIRT6+Scutellarin system, respectively. Figures S2 and S3 also shown the conformation difference of SIRT6 at SIRT6 system and SIRT6+inhibitor system. These data indicate that the conformation of SIRT6 is more compact in the presence of inhibitor systems than in the absence of inhibitor systems.

Overall, the analysis of the dynamics shows that our MD have reasonably converged and that the inhibitors have the same impact on the conformation of SIRT6.

**Figure 2:**
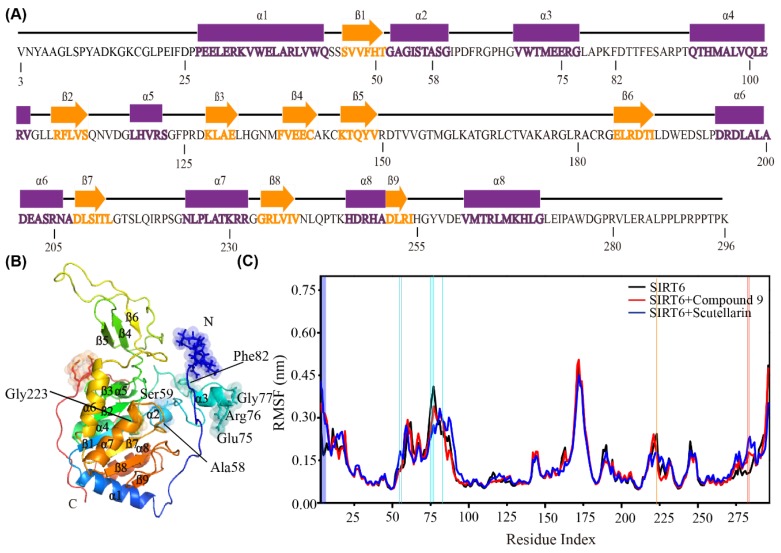
The effect of inhibitors on the flexibility of the residues. (**A**) The sequence of SIRT6 (PBD ID: 3K35). Strands are represented by orange arrows and helices are shown as purple cylinders. (**B**) The 3D structure of SIRT6 in the new cartoon representation. The flexible residues are shown as sticks and spheres. (**C**) The analysis of the average Cα root mean square fluctuations (RMSFs) of SIRT6 in the three systems. The big difference of RMSFs are symbolized with the light blue line (α2), cyan line (α3), orange line (α7), dark blue line (N terminus) and red line (C terminus) according to the domain of SIRT6 are given in vertical direction.

The inhibitors affect the flexibility of the residues and the interactions between the residues of SIRT6. To further describe the conformational characteristics of SIRT6 in the presence or absence of the inhibitor systems, we plotted the sequence of SIRT6 in Figure 2A and the 3D structure of SIRT6 in the new cartoon representation in Figure 2B and Figure S1A. SIRT6 consists of 9 β-strands and 8 helices, as shown in Figure 2B. This structure has been shown in Pan’s work [24]. To check the flexibility of each residue of SIRT6, we calculated the RMSF value of the alpha carbon for each residue of SIRT6 by removing the first 100 ns of each MD simulation. As shown in Figure 2C, the analysis of the RMSFs of SIRT6 is averaged over all the MD simulations for each system. Interestingly, we found that in addition to the residues at the N-terminus, residues Ala58, Ser59, Glu75, Arg76, Gly77 and Phe82 in the small domain [24] are also very flexible, as shown in Figure 2B,C, Table S1 and Figure S4. We can conclude that the residues in the small domain related to the long hydrophobic channel are more flexible than the other residues in the SIRT6 system. 

**Figure 3:**
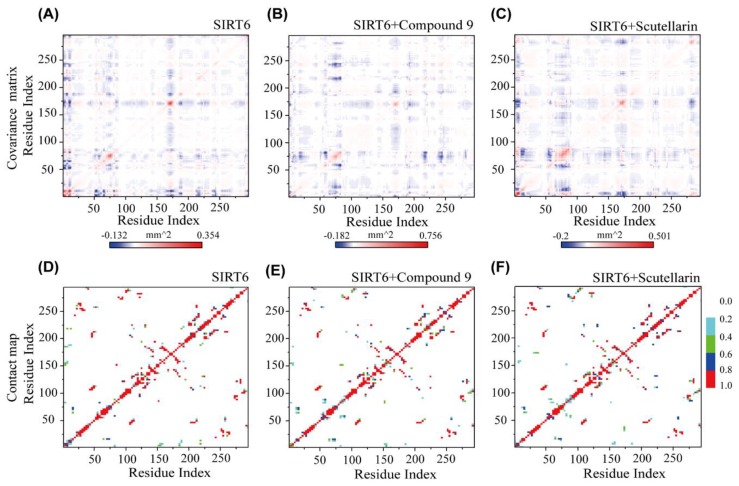
The effect of inhibitors on the interactions between the residues of SIRT6. Covariance matrix and residue–residue contact probability maps for SIRT6, respectively, in the SIRT6 system (**A**,**D**); in the SIRT6+Compound 9 system (**B**,**E**); and in the SIRT6+Scutellarin system (**C**,**F**).

Even with the large structural flexibility in the 2 complex systems, the inhibitors can retain a global residue–residue dynamic correlation as that is similar to SIRT6 in the single SIRT6 system. From Figure 3A, we can see that the fluctuating residues (residues 75–85 and residues 165–175) display the strong negative correlation with all the other residues. Interestingly, we found that residues 75–85 are near the hydrophobic channel and residues 165–175 belong to the small domain [24], as shown in Figure 2. As shown in Figure 3B,C residues 65–85 correlate well with residues 210–260, which are important for inhibitor binding [23]. From our data, we can conclude that the small domain has a negative correlation with the long channel, which is the binding site for the inhibitors of SIRT6.

To further understand the roles of the inhibitors on the dominant SIRT6 interactions and to estimate the residue-residue interactions of SIRT6, we plotted the contact probability between each pair of residues in the absence and presence of the inhibitors. As shown in Figure 3D–F, the residue–residue interaction patterns of the three systems are different. Without the inhibitors, the dominant peptide–peptide interactions occur between residues 75–120 and residues 271–296. However, the contacts between these residues become strong in the presence of the inhibitors in Figure 3E,F. Interestingly, most of these residue pairs display reduced contact probability in the SIRT6+Compound 9 and SIRT6+Scutellarin systems. Two key regions of SIRT6 play critical roles in molecular progress: the flexible C–terminal region [24] and the small domain region. 

**Figure 4:**
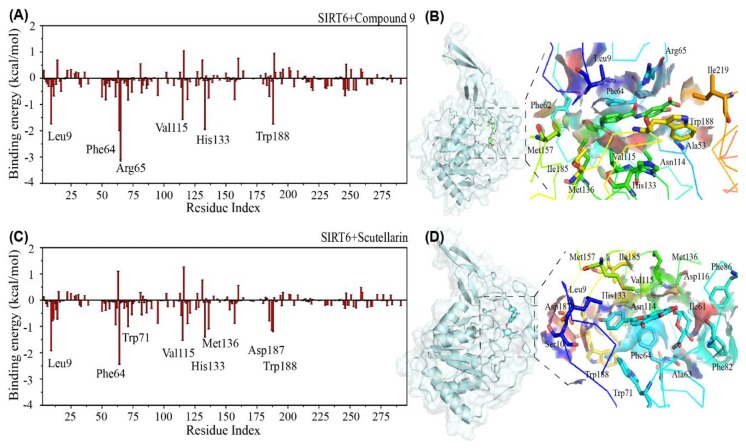
Residue-based binding site analysis. The binding free energy (in kcal/mol) between each residue of SIRT6 and the inhibitors (**A**,**C**); The binding modes between the inhibitors and SIRT6 are described in detail (**B**,**D**). The respective conformational snapshot is after 400 ns of each molecular dynamics (MD) simulation. The protein SIRT6 is shown in cartoon style and surface representation and the inhibitors are shown as sticks using PyMOL (**A**,**B**) for the SIRT6+Compound 9 system and (**C**,**D**) for the SIRT6+Scutellarin system.

Identification of the binding sites of SIRT6 by calculating the binding energies. After investigating the convergence of the research systems and the influence of the inhibitors on the conformation of SIRT6, we examined the binding free energy of the inhibitors bound to the protein SIRT6 using the MM/PBSA method. Interestingly, we found that the overall values are −25.66 kcal/mol (Compound 9) and −23.65 kcal/mol) (Scutellarin), respectively. The structurally similar inhibitors have the close bound energy with SIRT6. To understand the underlying interactive mechanism between the protein and the inhibitors, the first step is to identify the most favourable residues. To solve this problem, we first calculated the binding free energy of the inhibitors with each amino acid residue of SIRT6 by discarding the first 100 ns of the three MD trajectories with the MM/PBSA method. Figure 4A shows that Compound 9 has a preference for the long hydrophobic channel (residues: Leu9, Phe64, Arg65, Val115, His133 and Trp188). Interestingly, we found that the binding site of Scutellarin to SIRT6 (residues: Leu9, Asp63, Phe64, Trp71, Val115, Asp116, His133, Met136, Asp187 and Trp188) is very similar to the binding site of Compound 9. The experiments also reported the residues: Leu9, Phe82, Phe86 Val153 and Met157 are the binding sites located at the surface in the long SIRT6 channel pocket (shown in Figure S5) [43]. To provide an instructive view of the binding position distribution of the small molecules on the surface of SIRT6, we present the binding residues of SIRT6 around the small molecules in Figure 4B,D, Figures S6 and S7. From the snapshot, we can see that the small molecule Compound 9 and Scutellarin insert into the long channel pocket. It has been reported that the binding pocket is between the small domain and the larger Rossmann-fold domain [24].

In Tables S2 and S3, the values of less than −1.0 kcal/mol binding energy are listed and the values of more than 30% of the contact probability from the SIRT6+Compound 9 system and SIRT6+Scutellarin system. From Table S2, we can see that the hydrophobic residues Leu9, Phe64, Arg65, Val115, His133 and Trp188 play important roles in the interactions between Compound 9 and SIRT6. From Table S3, we can also see that the hydrophobic residues Leu9, Phe64, Trp71, Val115, His133, Met136, Asp187 and Trp188 play important roles in the interaction between the inhibitor Scutellarin and SIRT6. Most of the residues are distributed in the N–flexible domain and the small domain. As a result, we can summarize that the interactions between the inhibitors and SIRT6 are mainly hydrophobic interactions. These data in Tables S2 and S3 agree well with the results in Figure 2.

Interestingly, from Figure 4, we can conclude that the two conformationally similar inhibitors have the same binding mode with SIRT6. In the experiments, You et al. [23,25] and Lu et al. [43] reported the binding sites of potential inhibitors to SIRT6 (Phe64, Phe82 and Phe86 at N terminus). Our results agree well with the experiments. Hydrophobic interactions play important roles between the inhibitors and the residues of SIRT6. In previous computer simulation studies, hydrophobic interactions between small molecules and proteins have also been reported [44,45,46,47,48].

**Figure 5:**
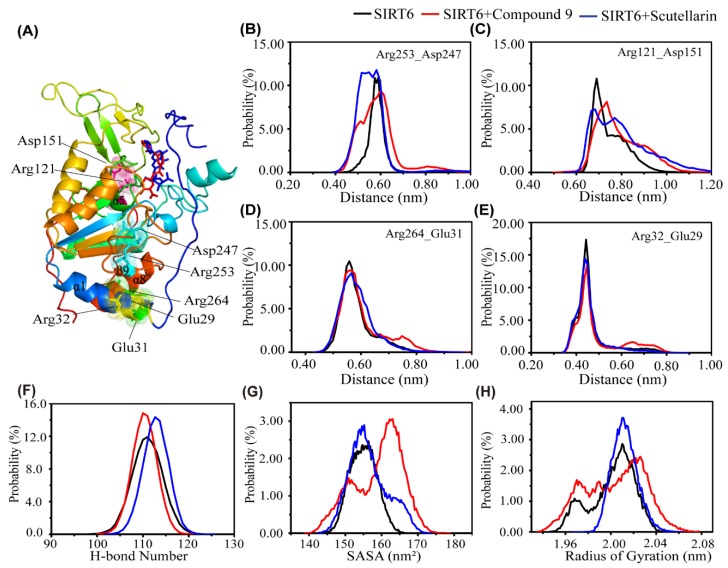
The influence of the inhibitors on the salt bridge and conformational properties of SIRT6. (**A**) Representative conformational snapshot of SIRT6. The SIRT6 protein is shown in cartoon style with the involved residues shown in surface representation and the inhibitors shown as sticks using PyMOL. The distance between Arg253 and Asp247 (**B**), Arg121 and Asp151 (**C**), Arg264 and Glu31 (**D**), Arg32 and Glu29 (**E**) in the SIRT6 (black), SIRT6+Compound 9 (red) and SIRT6+Scutellarin (blue) systems. (**F**) The probability distribution function for the number of H–bonds in SIRT6. (**G**) The probability distribution function of the SASA in SIRT6. (**H**)The probability distribution function of the radius of gyration in the SIRT6 system (black line), SIRT6+Compound 9 system (red line) and SIRT6+Scutellarin system (blue line).

The effect of different inhibitors on the conformation of SIRT6. To investigate the influence of the addition of small molecules on the salt bridge intuitively, we choose the typical salt bridges, which are distributed in the Rossmann–fold domain (Arg253-Asp247 and Arg121-Asp151), the N and C flexible domain (Arg264-Glu31) and the N-flexible domain (Arg32-Glu29). Interestingly, we found that the distance between Arg53 and Asp247 is quite different in the three systems. The peaks centre at 0.7 nm are a little higher in the presence of inhibitors than in the absence of an inhibitor, as shown in Figure 5C. However, we can also see that the peaks of minimum distance (Arg121-Asp151, Arg264-Glu31 and Arg32-Glu29) are located at almost 0.18 nm and there is little difference between the three systems. From our data, we can conclude that the influence of the small molecules on the salt bridge of SIRT6 is not obvious.

To further explore the influence of the inhibitors on the conformation of SIRT6, we calculated the probability distribution function of the number of H–bonds in SIRT6 in the three systems—SIRT6, SIRT6+Compound 9 and SIRT6+Scutellarin.As shown in Figure 5F, the peak for the number of H–bonds for SIRT6 was located at almost 110;however, this number was higher in the presence of inhibitors (15.5% in the SIRT6+Compound 9 system and 15.05% in the SIRT6+Scutellarin system) than in the absence of the inhibitors (10.0% in the SIRT6 system). To further examine the effect of the two different inhibitors on the conformation of SIRT6, we plotted the solvent accessible area (SASA) probability distribution using the data from the last 100 ns of the MD trajectory. Interestingly, we found that the SASA of SIRT6 displays one sharp peak at approximately 155 nm^2^ in the SIRT6 and SIRT6+Scutellarin systems and another peak at approximately 164 nm^2^ in the SIRT6+Compond 9 system. Figure 5H shows the probability of the radius of gyration (Rg). From Figure 5H, we can see that there is one sharp peak at approximately 2.01 nm in the SIRT6 and SIRT6+Scutellarin systems. In the SIRT6 and SIRT6+Scutellarin systems, the structure of SIRT6 is more compact than that in the SIRT6+Compond 9 system. Figure 5H agrees well with Figure 5F,G. These data indicate that the conformation of SIRT6 with small molecules is more compact than without small molecules. However, the two different inhibitors, Compound 9 and Scutellarin, have different effects on the formation of the compact state of SIRT6. 

**Figure 6:**
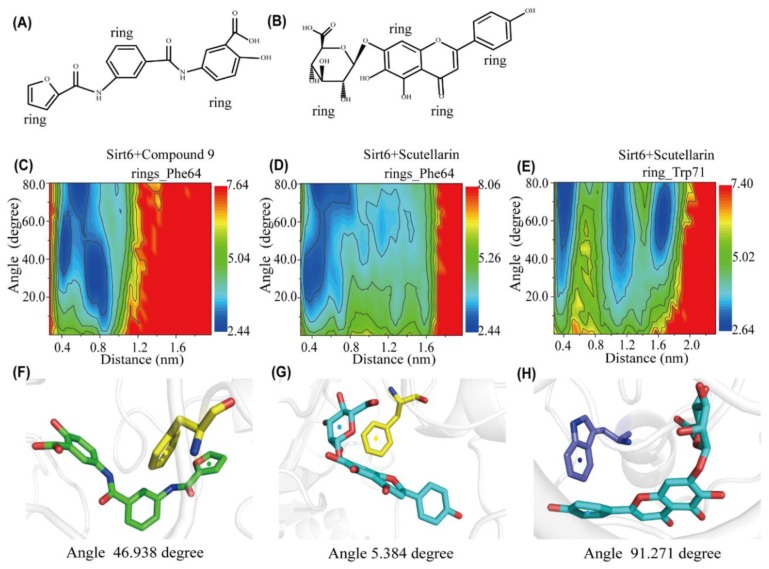
Analysis of aromatic stacking interactions between the rings of the inhibitors and aromatic residues. The chemical structures of Compound 9 (**A**) and Scutellarin (**B**). The free energy landscape as a function of the centroid distance and the angle between the rings of the inhibitors and the rings of aromatic residues of SIRT6 for the SIRT6+Compound 9 system (**C**,**F**) and SIRT6+Scutellarin system (**D**,**E**,**G**,**H**). Representative snapshots using PyMOL showing a herringbone–aligned stacking orientation between the aromatic ring of Phe64 and the ring of Compound 9 (**F**) in the SIRT6+Compound 9 system, a parallel–aligned aromatic stacking orientation between the aromatic ring of Phe64 and the rings of Scutellarin (**G**) in the SIRT6+Scutellarin system and a perpendicular–aligned stacking orientation between the aromatic ring of Trp71 and the ring of Scutellarin in the SIRT6+Scutellarin system (**H**).

To better understand the details of the stacking patterns of the benzene–like rings of the inhibitors and the aromatic residues of SIRT6, we plotted the free energy surface as a function of two reaction coordinates: the centroid distance and the angle between the rings of the aromatic residues Phe64 and Trp71 and their closest benzene-like ring of the inhibitors. We named each ring of each inhibitor in Figure 6A,B. As shown in Figure 6C, there are three minimum-energy basins centred at (angle, distance) values of (5° < angle < 90° and 0.4 nm < distance < 0.85 nm) and the representative snapshot is shown in Figure 6F, which corresponds to herringbone-aligned stacking interactions between aromatic residue Phe64 and one of the rings of Compound 9. In Figure 6D, there are two minimum–energy basins located at (angle, distance) values of (18° < angle < 90° and 0.4 nm < distance < 0.80 nm). The representative snapshot is shown in Figure 6G, which corresponds to parallel-aligned aromatic stacking patterns between aromatic residue Phe64 and one of the rings of Scutellarin. The perpendicular-aligned π-π stacking geometry is also very popular in the SIRT6+Scutellarin system, as shown in Figure 6G. Interestingly, we also found that there are three minimum–energy peaks that correspond to herringbone-stacking patterns and perpendicular-aligned stacking patterns between the aromatic residue Trp71 and the rings of Scutellarin. These parallel–aligned, herringbone–aligned [49] and perpendicular–aligned aromatic stacking interactions (shown in Figure S8) between benzene-like rings has also been reported in previous studies [50,51]. From our analysis, we can conclude that π-π interactions between the inhibitors and SIRT6 are important during the progress of the dynamic simulations. The two inhibitors, Compound 9 and Scutellarin, have different π-π stacking patterns with the aromatic residues of SIRT6.

## 3. Materials and Methods

### 3.1. Research Systems

To investigate the binding model of small molecules and SIRT6, three systems were studied: SIRT6, SIRT6+Compound 9 and SIRT6+Scutellarin. We obtained the protein from the Protein Data Bank (PDB) code 3K35 [23,24] and chose chain A to build the initial state of the SIRT6 system. This model is an X-ray diffraction structure and Pan et al. selected the 3K35 complex as the reference structure for in silico screens because of its higher resolution. And we used the Swiss model method to connect the miss residues of SIRT6 (3K35). The SIRT6+Compound 9 system includes chain A of 3K35 and small molecule Compound 9 [30]. Each system was placed in a rectangular box of SPC water molecules with a minimum distance to the water box wall of 1.0 nm for the molecular dynamics simulations. The total numbers of atoms for the three systems were 4619, 4660 and 4670 for SIRT6, SIRT6+Compound 9 and SIRT6+Scutellarin, respectively. The number of atoms for small molecules was 41 for Compound 9 and 51 for Scutellarin. 

SIRT6. The starting state of the protein SIRT6 system is shown in Figure S1A. SIRT6 is shown in cartoon representation. Because of the flexibility protein in the loop and terminal section, the crystal structure of SIRT6 (3K35) is not complete. Using the Swiss model method, we connected the missing residues [52,53], as shown in Figure S9.

SIRT6+Compound 9 System. The initial state of the complex of Compound 9 and SIRT6 is shown in Figure S1D. We chose Compound 9 as the inhibitor because it has significant selectivity for SIRT6 [30]. To place the small molecule at the active site of the protein SIRT6 [30], we chose Asn114 as the docking ligand residue of the complex.

SIRT6+Scutellarin System. The initial state of the complex of Scutellarin and SIRT6 is shown in Figure S1E. The reason we chose Scutellarin as the inhibitor is that it has a similar structure to Compound 9 [30] and is a potential therapeutic inhibitor for cancer from TCMS [33].

Therefore, the initial state of the protein in complex with SIRT6 is the same as in the SIRT6+Compound 9 system. Scutellarin was placed at a similar site to that of Compound 9, as shown in Figure S1E. The SIRT6+Scutellarin system is shown in the same representation as that of the SIRT6+Compound 9 system. The docking of the ligand Scutellarin with SIRT6 was similar to Compound 9 in the SIRT6+Compound 9 system. 

### 3.2. Molecular Docking Strategy

In order to screen the potential inhibitor of SIRT6 firstly, we used AutoDock, which is an automated tool for predicting the interaction of ligands with biomacromolecular targets. In a recent study, it was reported that the deprotonated form of SIRT6 favours the formation of hydrogen bonds with the amino group of Asn114 [30] and Asn114 is shown as a flexible residue in this case. We used semi-flexible docking, which is suitable for docking molecules of intermediate size. Eventually, AutoDock writes the coordinates in the docking log file for ten docked conformations along with information on the clustering and interaction energies [42,54]. In terms of the lowest energy and the correct position, we choose the most appropriate cluster.

### 3.3. MD Simulations

To gain deep insight into the interaction between the inhibitors and SIRT6 at an atomistic level, we performed molecular dynamics (MD) simulations.

In this work, we investigated the SIRT6, SIRT6+Compound 9 and SIRT6+Scutellarin conformations in NaCl solution conducting extensive atomistic MD simulations. To mimic the experimental neutral pH conditions, the residues of Asp, Glu, Arg and Lys were charged (Asp^−1^, Glu^−1^, Arg^+1^, Lys^+1^) and the N- and C-termini were also charged (NH_3_^+^, COO^−^). Counterions (Cl^−1^) were added to neutralize the three systems. The ionic (Cl^−1^) concentration is 0.042 mol/L. All MD simulations were performed in the isothermal-isobaric (NPT) ensemble with the GROMACS-2016.4 software package [55,56]. Recently, we found that the AMBER99SB force field has been widely used [44,57,58] in several computational studies of small molecule and protein complex systems [59,60]. The temperature was maintained close to 310 K by weak coupling to an external temperature bath with a coupling constant of 0.1 ps and the pressure was kept at 1 bar using a coupling time of 2.0 ps. The visual inspection of three systems was carefully performed using VMD software [61].

### 3.4. Analysis Methods

We performed the analysis using our in-house developed codes and the GROMACS facilities [46]. The MD trajectories were analysed using several parameters. First, we discarded the first 100 ns MD trajectory with the purpose of removing the bias of the initial states. The backbone root mean square deviation (RMSD), α-carbon root mean square fluctuations (RMSFs) and the solvent accessible surface area (SASA) were analysed in our study. The RMSD of SIRT6 was calculated with respect to the initial structure of SIRT6 which was obtained from 10 ns’ energy optimization. The binding probabilities between the protein SIRT6 and inhibitors were analysed using contact probability. Here, we identified that the residue is in contact with the inhibitors when the distance between the atoms of the inhibitor and residue of the protein was within 0.54 nm. In this study, a contact is defined when the minimum distance is less than 0.46 nm [62]. A hydrogen bond was considered to be formed when the N⋯O distance was within 3.5 Ǻ and the N-H⋯O angle was between 150° and 180° [51]. We also calculated the 2D free energy landscape, applying the formula, −RTlnH(x,y), where H(x,y) is the histogram of two selected reaction coordinates.

We calculated the binding energies between the protein SIRT6 and inhibitors by using the MM/PBSA method implemented in the GROMACS package [63,64,65]. In the MM/PBSA method, the binding free energy (ΔG_binding_) between a ligand and the receptor is ΔG_binding_ = ΔE_MM_ + ΔG_solv_ − TΔS, where ΔE_MM_ is the gas phase energy consisting of electrostatic (ΔE_elec_) and van der Waals (ΔE_vdW_) terms. ΔG_solv_ consists of the polar solvation energy, ΔG_polar_ and the nonpolar solvation component ΔG_surf_. ΔG_polar_ is calculated by the GB model [64] and ΔG_suf_ is estimated by the solvent accessible surface area (SASA). As the binding energy (ΔG_binding_) reported here is the relative binding free energy, the contribution of conformational entropy of the small molecule was ignored in accordance with a number of previous computational studies [45,46,66,67]. Therefore, in this study, we use the formula ΔG_binding_ = ΔE_MM_ + ΔG_solv_ [61] to calculate the binding free energy.

## 4. Conclusions

In summary, in this study, based on the conformational characteristics of the inhibitor Compound 9, we explored the natural molecule Scutellarin from the TCMS library as a candidate inhibitor of SIRT6. To characterize the inhibitory mechanism of inhibitor binding with SIRT6, we carried out nine all-atom MD simulations. Interestingly, we found that the structurally similar inhibitors, Compound 9 and Scutellarin, have similar effective interactions and binding sites with SIRT6. Hydrophobic and π–stacking interactions play important roles in the interactions between the inhibitors and SIRT6. Our results provide the interactional mechanism of SIRT6 and the inhibitors and provide Scutellarin as the new potential inhibitor of SIRT6. From our study, we can propose the hypotheses that the effectiveness of small molecules depends on the configuration matching between small molecules and proteins. In summary, our results reveal the interactive mechanism of SIRT6 and the inhibitors and we also provide Scutellarin as a new potential inhibitor of SIRT6.

## Figures and Tables

**Table 1:** The chemical structures of the potential inhibitors of SIRT6 selected from Traditional Chinese Medicines (TCMS) using AutoDock methods.

Compound	English Name	Chinese Herb Medicine	Structure Formula	Binding Energy (kcal/mol)
1	Puerarin	Ge Geng	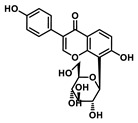	−7.05
2	Shikonofuran E	Zi Cao	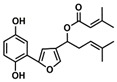	−6.1
3	Daidzin	Ge Geng	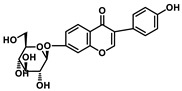	−7.93
4	Sophoricoside	Huai Hua	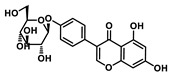	−6.73
5	Baohuoside I	Epimedium koreanum Nakai	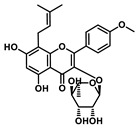	−6.71
6	Carthamin	Hong Hua	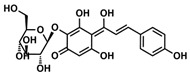	−6.14
7	Liquiritin	Cu Mao; Gan Cao	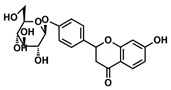	−7.74
8	Wogonoside	Huang Qin; Chuan Huang Qin; Dian Huang Qin	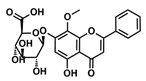	−6.92
9	BAS13555470 (Compound 9)	-	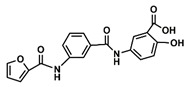	−8.66
10	Scutellarin	Ban Zhi Lian; Huang Qin	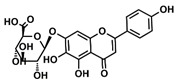	−9.43

## Supplementary Materials

Supplementary materials can be found at https://www.mdpi.com/1422-0067/21/7/2601/s1.

## References

[1] Finkel T., Deng C.-X., Mostoslavsky R. (2009). Recent progress in the biology and physiology of sirtuins. Nature.

[2] Kaeberlein M., McVey M., Guarente L. (1999). The SIR2/3/4 complex and SIR2 alone promote longevity in Saccharomyces cerevisiae by two different mechanisms. Genes Dev..

[3] Garcia-Peterson L.M., Guzmán-Pérez G., Krier C.R., Ahmad N. (2019). The sirtuin 6: An overture in skin cancer. Exp. Dermatol..

[4] Khan R.I., Nirzhor S.S.R., Akter R. (2018). A review of the recent advances made with SIRT6 and its implications on aging related processes, major human diseases and possible therapeutic targets. Biomolecules.

[5] Feldman J.L., Dittenhafer-Reed K.E., Denu J.M. (2012). Sirtuin Catalysis and Regulation. J. Biol. Chem..

[6] Chalkiadaki A., Guarente L. (2015). The multifaceted functions of sirtuins in cancer. Nat. Rev. Cancer.

[7] Jackson M.D., Denu J.M. (2002). Structural identification of 2′-and 3′-O-acetyl-ADP-ribose as novel metabolites derived from the Sir2 family of β-NAD+-dependent histone/protein deacetylases. J. Biol. Chem..

[8] Michan S., Sinclair D. (2007). Sirtuins in mammals: Insights into their biological function. Biochem. J..

[9] Ota H., Tokunaga E., Chang K., Hikasa M., Iijima K., Eto M., Kozaki K., Akishita M., Ouchi Y., Kaneki M. (2006). Sirt1 inhibitor, Sirtinol, induces senescence-like growth arrest with attenuated Ras–MAPK signaling in human cancer cells. Oncogene.

[10] Luthi-Carter R., Taylor D.M., Pallos J., Lambert E., Amore A., Parker A., Moffitt H., Smith D.L., Runne H., Gokce O. (2010). SIRT2 inhibition achieves neuroprotection by decreasing sterol biosynthesis. Proc. Natl. Acad. Sci. USA.

[11] Schuetz A., Min J., Antoshenko T., Wang C.-L., Allali-Hassani A., Dong A., Loppnau P., Vedadi M., Bochkarev A., Sternglanz R. (2007). Structural basis of inhibition of the human NAD+-dependent deacetylase SIRT5 by suramin. Structure.

[12] Bruzzone S., Daniele Parenti M., Grozio A., Ballestrero A., Bauer I., Del Rio A., Nencioni A. (2013). Rejuvenating Sirtuins: The Rise of a New Family of Cancer Drug Targets. Curr. Pharm. Des..

[13] Kawahara T.L., Michishita E., Adler A.S., Damian M., Berber E., Lin M., McCord R.A., Ongaigui K.C., Boxer L.D., Chang H.Y. (2009). SIRT6 links histone H3 lysine 9 deacetylation to NF-kappaB-dependent gene expression and organismal life span. Cell.

[14] Kugel S., Mostoslavsky R. (2014). Chromatin and beyond: The multitasking roles for SIRT6. Trends Biochem. Sci..

[15] Michishita E., McCord R.A., Berber E., Kioi M., Padilla-Nash H., Damian M., Cheung P., Kusumoto R., Kawahara T.L., Barrett J.C. (2008). SIRT6 is a histone H3 lysine 9 deacetylase that modulates telomeric chromatin. Nature.

[16] Michishita E., McCord R.A., Boxer L.D., Barber M.F., Hong T., Gozani O., Chua K.F. (2009). Cell cycle-dependent deacetylation of telomeric histone H3 lysine K56 by human SIRT6. Cell Cycle.

[17] Mostoslavsky R., Chua K.F., Lombard D.B., Pang W.W., Fischer M.R., Gellon L., Liu P., Mostoslavsky G., Franco S., Murphy M.M. (2006). Genomic instability and aging-like phenotype in the absence of mammalian SIRT6. Cell.

[18] Yang B., Zwaans B.M., Eckersdorff M., Lombard D.B. (2009). The sirtuin SIRT6 deacetylates H3 K56Ac in vivo to promote genomic stability. Cell Cycle.

[19] Zhong L., D’Urso A., Toiber D., Sebastian C., Henry R.E., Vadysirisack D.D., Guimaraes A., Marinelli B., Wikstrom J.D., Nir T. (2010). The histone deacetylase Sirt6 regulates glucose homeostasis via Hif1alpha. Cell.

[20] Huang N., Liu Z., Zhu J., Cui Z., Li Y., Yu Y., Sun F., Pan Q., Yang Q. (2017). Sirtuin 6 plays an oncogenic role and induces cell autophagy in esophageal cancer cells. Tumour Biol..

[21] Khongkow M., Olmos Y., Gong C., Gomes A.R., Monteiro L.J., Yagüe E., Cavaco T.B., Khongkow P., Man E.P.S., Laohasinnarong S. (2013). SIRT6 modulates paclitaxel and epirubicin resistance and survival in breast cancer. Carcinogenesis.

[22] Ming M., Han W., Zhao B., Sundaresan N.R., Deng C.-X., Gupta M.P., He Y.-Y. (2014). SIRT6 promotes COX-2 expression and acts as an oncogene in skin cancer. Cancer Res..

[23] You W., Steegborn C. (2018). Structural Basis of Sirtuin 6 Inhibition by the Hydroxamate Trichostatin A: Implications for Protein Deacylase Drug Development. J. Med. Chem..

[24] Pan P.W., Feldman J.L., Devries M.K., Dong A., Edwards A.M., Denu J.M. (2011). Structure and biochemical functions of SIRT6. J. Biol. Chem..

[25] You W., Zheng W., Weiss S., Chua K.F., Steegborn C. (2019). Structural basis for the activation and inhibition of Sirtuin 6 by quercetin and its derivatives. Sci. Rep..

[26] Hanukoglu I. (2015). Proteopedia: Rossmann fold: A beta-alpha-beta fold at dinucleotide binding sites. Biochem. Mol. Biol. Educ..

[27] Huang Z., Zhao J., Deng W., Chen Y., Shang J., Song K., Zhang L., Wang C., Lu S., Yang X. (2018). Identification of a cellularly active SIRT6 allosteric activator. Nat. Chem. Biol..

[28] Alcaín F.J., Minor R.K., Villalba J.M., de Cabo R., Fahy G.M., West M.D., Harris S.B. (2010). The Future of Aging.

[29] You W., Rotili D., Li T.-M., Kambach C., Meleshin M., Schutkowski M., Chua K.F., Mai A., Steegborn C. (2017). Structural Basis of Sirtuin 6 Activation by Synthetic Small Molecules. Angew. Chem. Int. Ed..

[30] Parenti M.D., Grozio A., Bauer I., Galeno L., Damonte P., Millo E., Sociali G., Franceschi C., Ballestrero A., Bruzzone S. (2014). Discovery of novel and selective SIRT6 inhibitors. J. Med. Chem..

[31] Damonte P., Sociali G., Parenti M.D., Soncini D., Bauer I., Boero S., Grozio A., Holtey M.V., Piacente F., Becherini P. (2017). SIRT6 inhibitors with salicylate-like structure show immunosuppressive and chemosensitizing effects. Bioorg. Med. Chem..

[32] Commission C.P. (2010). Pharmacopoeia of the People’s Republic of China Part 1.

[33] Goh D., Lee Y.H., Ong E.S. (2005). Inhibitory effects of a chemically standardized extract from Scutellariabarbata in human colon cancer cell lines, LoVo. J. Agric. Food Chem..

[34] Kawser Hossain M., AbdalDayem A., Han J., Yin Y., Kim K., Kumar Saha S., Yang G.M., Choi H.Y., Cho S.G. (2016). Molecular Mechanisms of the Anti-Obesity and Anti-Diabetic Properties of Flavonoids. Int. J. Mol. Sci..

[35] Prasain J.K., Carlson S.H., Wyss J.M. (2010). Flavonoids and age-related disease: Risk, benefits and critical windows. Maturitas.

[36] Cook N. (1996). Flavonoids—Chemistry, metabolism, cardioprotective effects and dietary sources. J. Nutr. Biochem..

[37] Lee E.-R., Kang Y.-J., Choi H.-Y., Kang G.-H., Kim J.-H., Kim B.-W., Han Y.S., Nah S.-Y., Paik H.-D., Park Y.-S.J.B. (2007). Induction of apoptotic cell death by synthetic naringenin derivatives in human lung epithelial carcinoma A549 cells. Biol. Pharm. Bull..

[38] Ahn S.-Y., Jo M.S., Lee D., Baek S.-E., Baek J., Yu J.S., Jo J., Yun H., Kang K.S., Yoo J.-E. (2019). Dual effects of isoflavonoids from Puerarialobata roots on estrogenic activity and anti-proliferation of MCF-7 human breast carcinoma cells. Bioorg. Chem..

[39] Liu H., Zhu Y., Zhang T., Zhao Z., Zhao Y., Cheng P., Li H., Gao H., Su X. (2013). Anti-Tumor Effects of Atractylenolide I Isolated from Atractylodesmacrocephala in Human Lung Carcinoma Cell Lines. Molecules.

[40] Yin X., Zhou J., Jie C., Xing D., Zhang Y. (2004). Anticancer activity and mechanism of Scutellariabarbata extract on human lung cancer cell line A549. Life Sci..

[41] Chen W., Guang-ru X., Yu-rong S. (2003). Study on the anti-tumor effect in vivo of Glycyrrhizia polysaccharide and its mechanism. Chin. Clin. Oncol..

[42] Morris G.M., Huey R., Lindstrom W., Sanner M.F., Belew R.K., Goodsell D.S., Olson A.J. (2009). AutoDock4 and AutoDockTools4: Automated docking with selective receptor flexibility. J. Comput. Chem..

[43] Lu S., He X., Ni D., Zhang J. (2019). Allosteric Modulator Discovery: From Serendipity to Structure-Based Design. J. Med. Chem..

[44] Sun Y., Qian Z., Wei G. (2016). The inhibitory mechanism of a fullerene derivative against amyloid-beta peptide aggregation: An atomistic simulation study. Phys. Chem. Chem. Phys..

[45] Song M., Sun Y., Luo Y., Zhu Y., Liu Y., Li H. (2018). Exploring the Mechanism of Inhibition of Au Nanoparticles on the Aggregation of Amyloid-β(16-22) Peptides at the Atom Level by All-Atom Molecular Dynamics. Int. J. Mol. Sci..

[46] Song M., Zhu Y., Wei G., Li H. (2017). Carbon nanotube prevents the secondary structure formation of amyloid-β trimers: An all-atom molecular dynamics study. Mol. Simul..

[47] Jin Y., Sun Y., Lei J., Wei G. (2018). Dihydrochalcone molecules destabilize Alzheimer’s amyloid-β protofibrils through binding to the protofibril cavity. Phys. Chem. Chem. Phys..

[48] Ge X., Sun Y., Ding F. (2018). Structures and dynamics of β-barrel oligomer intermediates of amyloid-beta16-22 aggregation. Biochim. Biophys. Acta.

[49] Zou Y., Qian Z., Chen Y., Qian H., Wei G., Zhang Q. (2019). Norepinephrine Inhibits Alzheimer’s Amyloid-β Peptide Aggregation and Destabilizes Amyloid-β Protofibrils: A Molecular Dynamics Simulation Study. ACS Chem. Neurosci..

[50] Li H., Luo Y., Derreumaux P., Wei G. (2011). Carbon Nanotube Inhibits the Formation of β-Sheet-Rich Oligomers of the Alzheimer’s Amyloid-β(16-22) Peptide. Biophys. J..

[51] Mo Y., Brahmachari S., Lei J., Gilead S., Tang Y., Gazit E., Wei G. (2018). The inhibitory effect of hydroxylated carbon nanotubes on the aggregation of human islet amyloid polypeptide revealed by a combined computational and experimental study. ACS Chem. Neurosci..

[52] Bienert S., Waterhouse A., de Beer T.A.P., Tauriello G., Studer G., Bordoli L., Schwede T. (2017). The SWISS-MODEL Repository—New features and functionality. Nucleic Acids Res..

[53] Bertoni M., Kiefer F., Biasini M., Bordoli L., Schwede T. (2017). Modeling protein quaternary structure of homo-and hetero-oligomers beyond binary interactions by homology. Sci. Rep..

[54] Forli S., Olson A.J. (2012). A force field with discrete displaceable waters and desolvation entropy for hydrated ligand docking. J. Med. Chem..

[55] Hess B., Kutzner C., Van Der Spoel D., Lindahl E. (2008). GROMACS 4: Algorithms for highly efficient, load-balanced and scalable molecular simulation. J. Chem. Theory Comput..

[56] Lindorff-Larsen K., Piana S., Palmo K., Maragakis P., Klepeis J.L., Dror R.O., Shaw D.E. (2010). Improved side-chain torsion potentials for the Amber ff99SB protein force field. Proteins Struct. Funct. Bioinform..

[57] Guan X., Lin P., Knoll E., Chakrabarti R. (2014). Mechanism of inhibition of the human sirtuin enzyme SIRT3 by nicotinamide: Computational and experimental studies. PLoS ONE.

[58] Karaman B., Sippl W. (2015). Docking and binding free energy calculations of sirtuin inhibitors. Eur. J. Med. Chem..

[59] Wang J., Cieplak P., Kollman P.A. (2000). How well does a restrained electrostatic potential (RESP) model perform in calculating conformational energies of organic and biological molecules?. J. Comput. Chem..

[60] Hornak V., Abel R., Okur A., Strockbine B., Roitberg A., Simmerling C. (2006). Comparison of multiple Amber force fields and development of improved protein backbone parameters. Proteins.

[61] Xu Y., Zheng Q., Yu L., Zhang H., Sun C. (2012). A molecular dynamics and computational study of human KAT3 involved in KYN pathway. Sci. China Chem..

[62] Day R., Bennion B.J., Ham S., Daggett V. (2002). Increasing Temperature Accelerates Protein Unfolding Without Changing the Pathway of Unfolding. J. Mol. Biol..

[63] Onufriev A., Bashford D., Case D.A. (2004). Exploring protein native states and large-scale conformational changes with a modified generalized born model. Proteins.

[64] Onufriev A., Bashford D., Case D.A. (2000). Modification of the generalized Born model suitable for macromolecules. J. Phys. Chem. B.

[65] Salomon-Ferrer R., Case D.A., Walker R.C. (2013). An overview of the Amber biomolecular simulation package. Wiley Interdiscip. Rev. Comput. Mol. Sci..

[66] Berhanu W.M., Hansmann U.H. (2013). The stability of cylindrin beta-barrel amyloid oligomer models-a molecular dynamics study. Proteins.

[67] Zhang T., Zhang J., Derreumaux P., Mu Y. (2013). Molecular mechanism of the inhibition of EGCG on the Alzheimer Abeta(1-42) dimer. J. Phys. Chem. B.

