# Supplementary material for: Structural Insight into the Interactions between Structurally Similar Inhibitors and SIRT6
Source: Int J Mol Sci. 2020 Apr 9;21(7):2601. doi: 10.3390/ijms21072601 (PMC7178056; doi:10.3390/ijms21072601)
Supplement: Supplementary file 1 [file ijms-21-02601-s001.pdf]

International Journal of *Molecular Sciences*

MDPI

Table S1. The average Cα root mean square fluctuation (RMSF) analysis of each residue and the difference in RMSF between the residues of SIRT6 in the three systems.

Residue Name	RMSF (nm)				
	SIRT6	SIRT6+Compound 9	SIRT6+Scutellarin	ΔColumn1-Column2	ΔColumn1-Column3
Val3	0.3209	0.3499	0.4266	-0.0291	-0.1057
Asn4	0.2054	0.3203	0.3953	-0.1149	-0.1899
Tyr5	0.1682	0.3196	0.3207	-0.1515	-0.1526
Ala6	0.1832	0.3122	0.2796	-0.1290	-0.0964
Ala7	0.2100	0.2913	0.2770	-0.0813	-0.0670
Ala58	0.1408	0.2131	0.2423	-0.0724	-0.1016
Ser59	0.1998	0.2606	0.2729	-0.0608	-0.0731
Glu75	0.3353	0.2547	0.2310	0.0805	0.1043
Arg76	0.3755	0.2925	0.2472	0.0830	0.1283
Gly77	0.4100	0.3405	0.2741	0.0695	0.1359
Phe82	0.2420	0.3001	0.3088	-0.0581	-0.0668
Gly223	0.2433	0.1700	0.1770	0.0733	0.0663
Glu283	0.1051	0.1786	0.2209	-0.0736	-0.1159
Arg284	0.1072	0.1752	0.2336	-0.0680	-0.1264

Table S2. The contact probability (%) and binding energies (kcal/mol) between the residues of SIRT6 and the small molecule Compound 9.

Residue Name	Binding Energy (kcal/mol)	Contact Probability (%)	Standard Error (Contact Probability)
Leu9	-1.74	98.97%	0.008
Phe64	-2.00	99.79%	0.002
Arg65	-3.14	53.89%	0.291
Val115	-1.57	99.45%	0.000
His133	-1.95	80.13%	0.000
Trp188	-1.75	96.64%	0.000

Table S3. The contact probability (%) and binding energies (kcal/mol) between the residues of SIRT6 and the small molecule Scutellarin.

Residue Name	Binding Energy (kcal/mol)	Contact Probability (%)	Standard Error (Contact Probability)
Leu9	-1.93	88.19%	0.110
Phe64	-2.44	98.84%	0.011
Trp71	-1.01	87.27%	0.082
Val115	-1.53	69.90%	0.011
His133	-1.41	32.99%	0.000
Met136	-1.10	77.33%	0.000
Asp187	-1.15	49.11%	0.001
Trp188	-1.20	30.24%	0.000

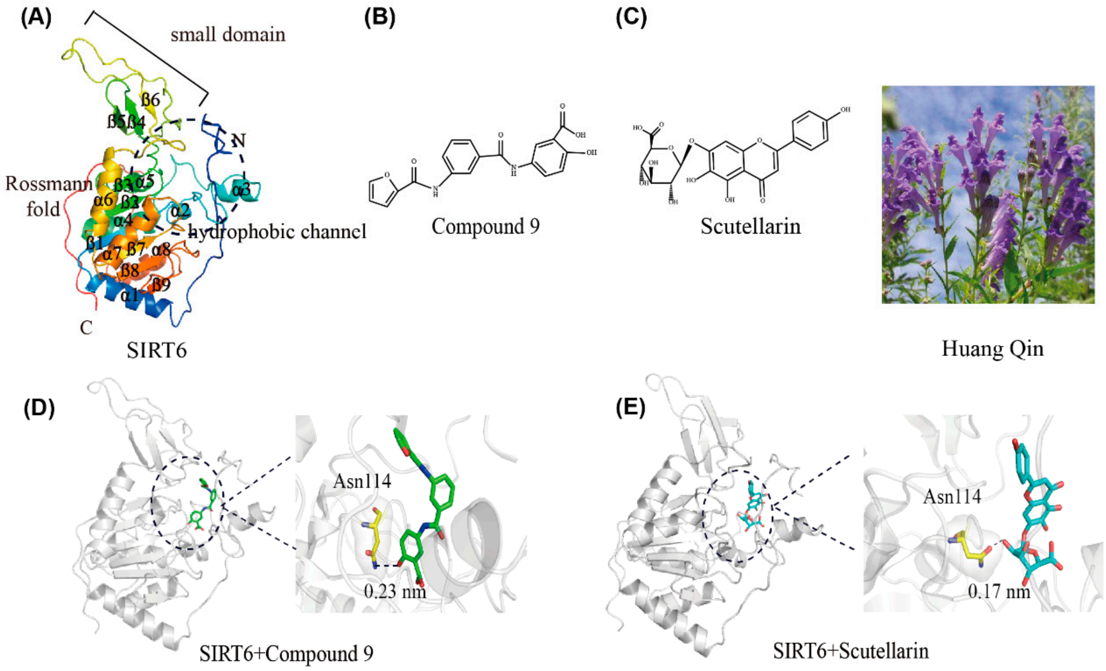

Figure S1. The initial structures of three systems. The 3D structure of SIRT6 in the new cartoon representation. (SIRT6 system) (**A**); the chemical structures of Compound 9 (**B**) and Scutellarin (**C**); the initial structure of the SIRT6+Compound 9 system (**D**) and SIRT6+Scutellarin system (**E**). SIRT6 is shown in cartoon representation, and the inhibitors are shown as sticks in the complex systems. The black dotted line circled the hydrophobic channel.

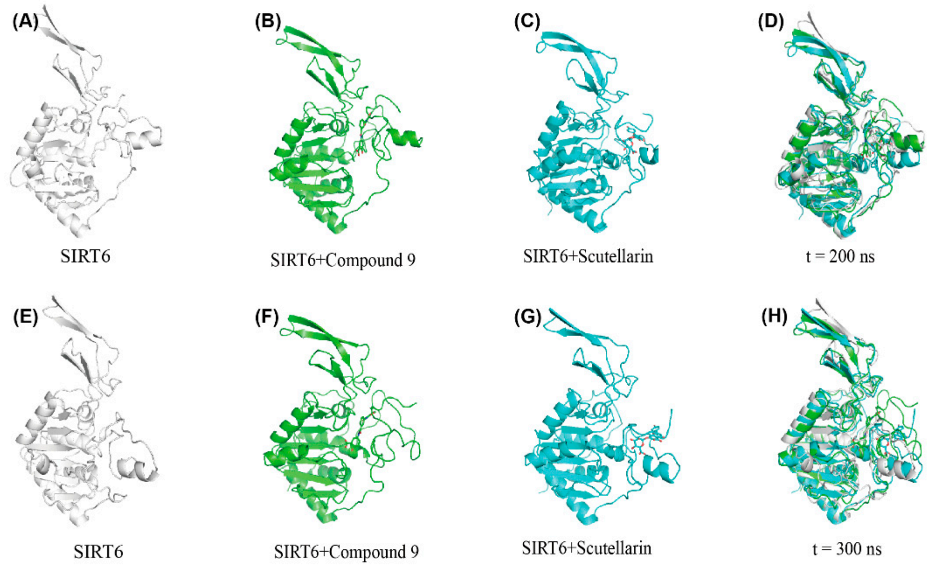

Figure S2. The 3D structure of SIRT6 in the new cartoon representation. (SIRT6 system) (**A**); the structures of SIRT6+Compound 9 (**B**) and SIRT6+Scutellarin (**C**); the overlap of SIRT6 in the three systems (**D**) at 200 ns. SIRT6 (**E**) the structures of SIRT6+Compound 9 (**F**) and SIRT6+Scutellarin (**G**); the overlap of SIRT6 in the three systems (**H**) at 300 ns. SIRT6 is shown in cartoon representation, and the inhibitors are shown as sticks in the complex systems.

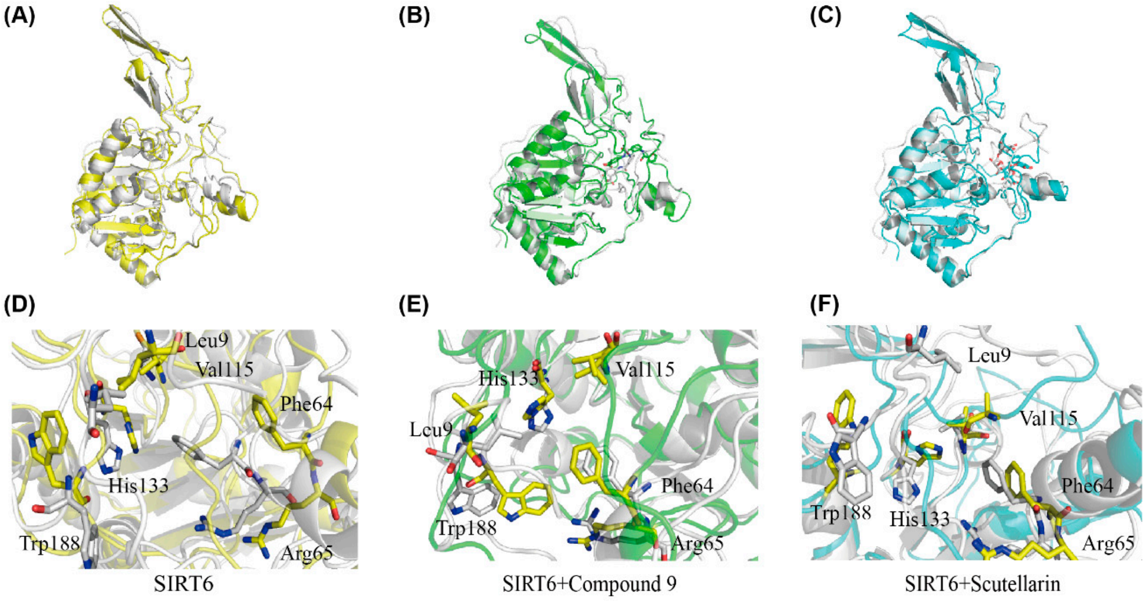

Figure S3. The 3D structure of SIRT6 in the new cartoon representation at the max RMSD peaks. the structures of (SIRT6 system) at 339.180 ns (**A**); the structures of SIRT6+Compound 9 at 350.96 ns (**B**) and SIRT6+Scutellarin at 388.39 ns (**C**); the conformational snapshot of systems are described in detail (**D**), (**E**), (**F**). SIRT6 is shown in cartoon representation, and the inhibitors are shown as sticks in the complex systems. The initial structure is shown in white of three systems.

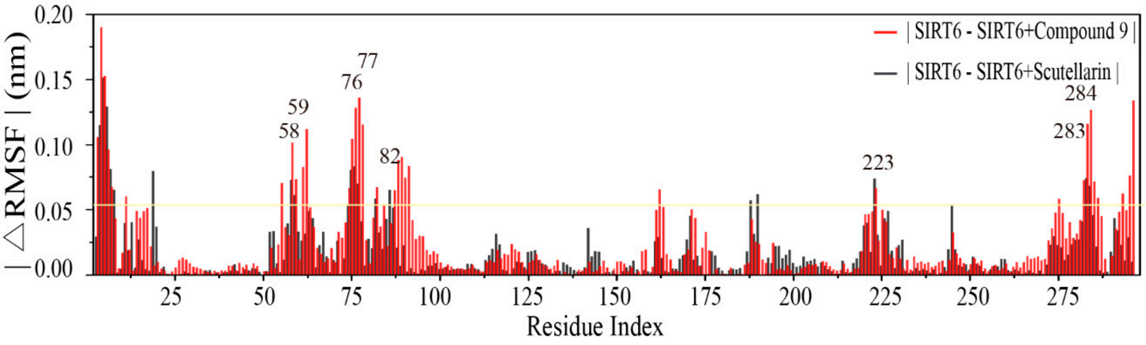

Figure S4. The absolute difference of Cα root mean square fluctuations (RMSF) between residues of SIRT6 in the three systems.

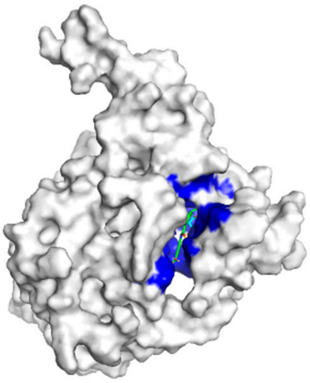

Figure S5. The representation of SIRT6 with small molecule in surface method. The hydrophobic channel is shown in blue colour.

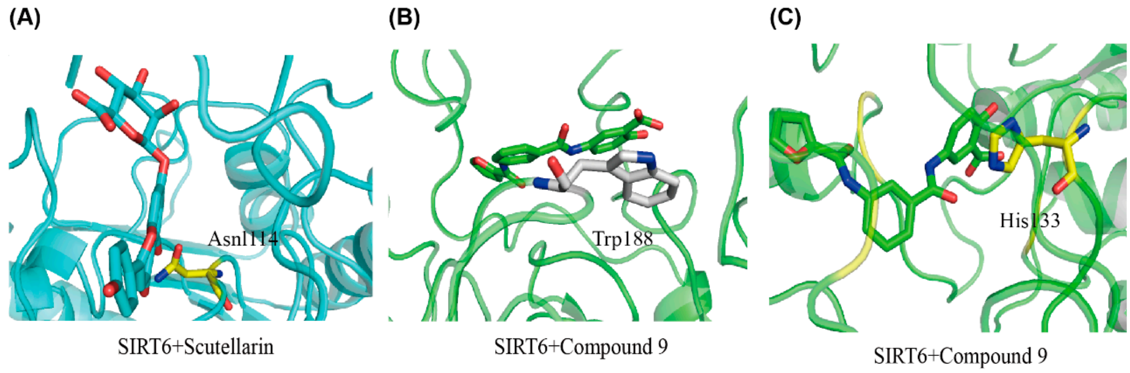

Figure S6. The conformational snapshot of interactions between compounds and SIRT6. The residues are coloured in yellow which are not hydrophobic. SIRT6 is shown in cartoon representation, and the inhibitors are shown as sticks in the complex systems.

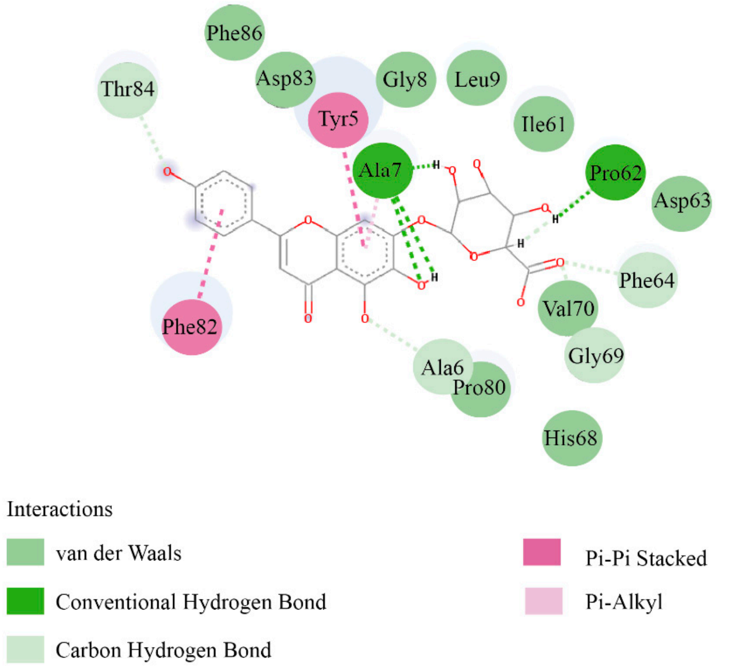

Figure S7. The binding model of SIRT6 and compounds.

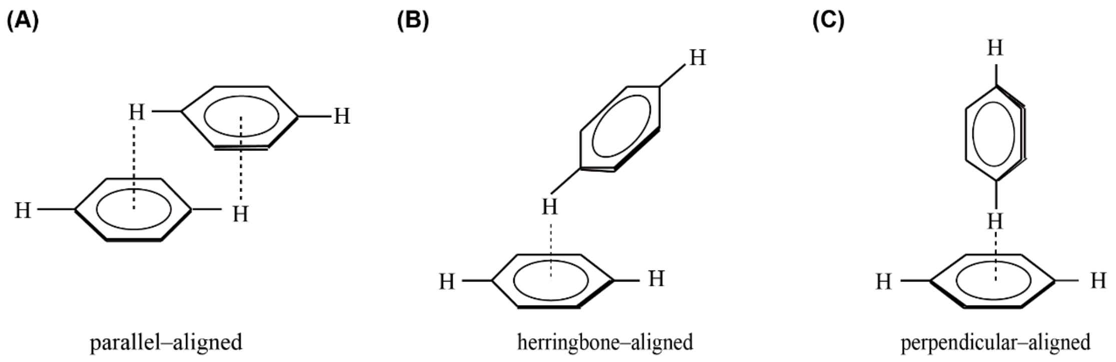

Figure S8. The typical π-π stacking interactions. (**A**) parallel-aligned π-π stacking interactions, (**B**) herringbone-alignedπ-π stacking interactions, (**C**) perpendicular-aligned π-π stacking interactions.

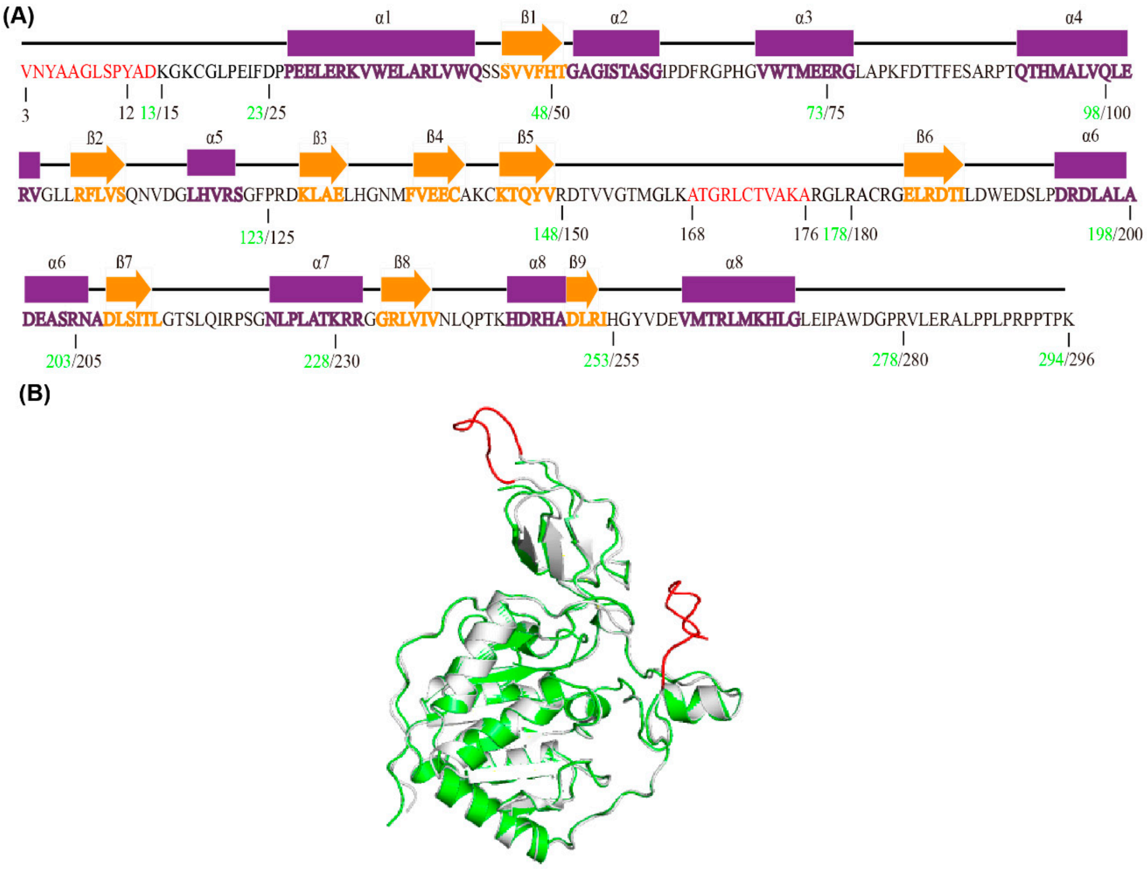

Figure S9. The difference between the crystal structure and the connected structure of SIRT6. (**A**) The sequence of SIRT6. The Number of sequence of crystal structure SIRT6 (3K35) are labelled in green colour. The Number of sequence of connected SIRT6 are labelled in black colour. (**B**)The connected SIRT6 is shown white. The original 3K35'chain A is green, the missing residues (Val3~Asp14, Ala169~Ala176) are shown red.
